# Editorial: Towards 2030: sustainable development goal 5: gender equality. A sociological perspective

**DOI:** 10.3389/fsoc.2023.1248990

**Published:** 2023-07-24

**Authors:** Anelise Gregis Estivalet, Gabriel Dvoskin

**Affiliations:** ^1^UNESCO Chair in Distance Education, University of Brasilia, Brasilia, Brazil; ^2^Linguistics and Literature Department, University of Buenos Aires, Buenos Aires, Argentina

**Keywords:** sustainable development, gender equality, goal 5, 2030 agenda, sociology

Historically, patriarchal systems have culturally emphasized the fragility and inferiority of women. Women were responsible for all household chores, and, in some cases, there were restrictions on their access to public spaces. Marginalized women were more vulnerable to domestic violence and private homes often constituted a space of deprivation – of public life, rights, and power. In the 1960s, women's movements began to develop from the construction of a collective female identity that gave them extraordinary importance in conjugating the relationship between the individual and the collective, between the public and the private. Since then, there has been a greater concern with the challenge of injustice, inequality, and non-recognition. By constituting women as the target of policies and understanding their performance as a differential in everyday relationships, the position of women in a situation of domination becomes public. There have been many achievements in recent decades, but progress is still needed in many instances.

As highlighted in the latest progress report on the United Nations Sustainable Development Goals (SDGs), equal participation in decision-making is still far from being achieved. According to the Sustainable Development Goals Report, the social and economic impacts of the COVID-19 pandemic have negatively affected progress toward gender equality. Violence against women and girls has intensified; child marriage, on the decline in recent years, is on the rise again; and women suffered a disproportionate share of job losses and increased care work at home. The pandemic has highlighted the need to act quickly to address pervasive global gender inequalities.

In this sense, this Research Topic sought to address the fifth goal of sustainable development, from a sociological paradigm, focusing on issues such as the impacts, whether economic or social, on progress toward gender equality, and on topics such as violence against women and girls, child marriage, sexual violence, including human trafficking, sexual exploitation and abuse, labor market and care work, public/private relationships, as well as decolonial and postcolonial gender studies. The Research Topic brings together critical contributions on the multidimensional poverty of rural women, on the African Gender and Development Index (AGDI), on domestic violence against women and on violence against trans people. Thus, the Research Topic “Towards 2030: sustainable development goal 5: gender equality. A sociological perspective” intends to contribute to the discussions about the 2030 agenda, as well as problematizes the challenges imposed to achieve gender equality.

The articles focus on gender issues, particularly on topics that address women and transgender people. Three main themes emerged: poverty, development and violence against women and transgender people. One of the main issues discussed is the persistent economic and gender inequality that affects rural women, a population that is often deprived of rights and information. Gender equality was also discussed in the article on the African Gender and Development Index and in research on victimization of violence against women in China and against transgender people in Bangladesh.

Barua and Khan, in the article “*Addressing violence against transgender people in Bangladesh: A call for policy intervention*” focuses on violence against transgender people and the violation of rights in Bangladesh, in addition to examining the types of violence against the transgender population and the actors who should be involved in resolving this situation. The study conducted by Wang and Sekiyama collected data from 412 women in Beijing and Shanghai, problematizing the issue of domestic violence against Chinese women who belong to different economic strata. In “Rural women's multidimensional relative poverty: measurement, dynamics, and influencing factors in China”, Peng concludes that education level, physical health, ideology, and family status are the main factors that affect women's multidimensional relative poverty in China. Charmes et al. assess the implementation of the African Gender and Development Index (AGDI) in the context of other gender indices, as well as discuss the latest revisions made.

Finally, the articles suggest that research can envision discussions about the victimization of women and transgender people from different social classes, as well as highlighting the importance of academic studies, the development of tools and public policies aimed at these issues. In addition, they alert to the main factors that contribute to the continuity of gender inequality.

To finish, we thank all the researchers who shared their interesting studies, believing that this Research Topic can contribute to the 2030 Agenda and, in particular, to the fifth Sustainable Development Goal, which is “Achieve gender equality and empower all women and girls.”

## Author contributions

All authors listed have made a substantial, direct, and intellectual contribution to the work and approved it for publication.

